# Identification of a *Campylobacter jejuni*-secreted protein required for maximal invasion of host cells

**DOI:** 10.1111/j.1365-2958.2009.06797.x

**Published:** 2009-07-27

**Authors:** Jeffrey E Christensen, Sophia A Pacheco, Michael E Konkel

**Affiliations:** Department of Microbiology, School of Molecular Biosciences, Washington State UniversityPullman, Washington, USA.

## Abstract

The food-borne pathogen *Campylobacter jejuni* is dependent on a functional flagellum for motility and the export of virulence proteins that promote maximal host cell invasion. Both the flagellar and non-flagellar proteins exported via the flagellar type III secretion system contain a sequence within the amino-terminus that directs their export from the bacterial cell. Accordingly, we developed a genetic screen to identify *C. jejuni* genes that encode a type III secretion amino-terminal sequence that utilizes the flagellar type III secretion system of *Yersinia enterocolitica* and a phospholipase reporter (*yplA*). We screened a library of 321 *C. jejuni* genes and identified proteins with putative type III secretion amino-terminal sequences. One gene identified by the screen was *Cj1242.* We generated a mutation in *Cj1242*, and performed growth rate, motility, secretion and INT 407 cell adherence and internalization assays. The *C. jejuni Cj1242* mutant was not altered in growth rate or motility when compared with the wild-type strain, but displayed an altered secretion profile and a reduction in host cell internalization. Based on the phenotype of the *C. jejuni Cj1242* mutant, we designated the protein *Campylobacter* invasion antigen C (CiaC). Collectively, our findings indicate that CiaC is a potentially important virulence factor.

## Introduction

*Campylobacter jejuni*, a Gram-negative pathogen, is one of the leading bacterial causes of gastroenteritis worldwide (Allos, 2001; Westrell *et al*., 2009). The clinical presentation of *C. jejuni-*mediated disease varies from one individual to another, where some individuals have watery diarrhoea and others experience diarrhoea with blood (Blaser *et al*., 1983; Friedman *et al*., 2004). The reason for the variation in clinical presentation is not known. We speculate that both the unique virulence factors of the infecting strain as well as the host innate immune response influence the presentation and severity of disease (Larson *et al*., 2008). The most severe form of campylobacteriosis, which is characterized by fever, severe abdominal cramps and diarrhoea containing blood and leucocytes, likely results from *C. jejuni* invasion of the intestinal epithelium. Indeed, intracellular bacteria have been observed by electron microscopy examination of samples from *C. jejuni*-infected individuals with acute infectious colitis characterized by diarrhoea with blood (van Spreeuwel *et al*., 1985).

*Campylobacter jejuni* must be metabolically active and secrete proteins from the flagellar type III secretion system (T3SS) for maximal invasion of host epithelial cells (Konkel and Cieplak, 1992; Konkel *et al*., 1993; 2004). The proteins synthesized and secreted by *C. jejuni* upon cocultivation with epithelial cells are termed *Campylobacter* invasion antigens (Cia) (Konkel *et al*., 1999). The importance of the Cia proteins in *C. jejuni* pathogenesis has been demonstrated with a *ciaB* mutant, which is deficient in Cia protein secretion. The severity and time of onset of disease in piglets inoculated with a *C. jejuni ciaB* null mutant is significantly attenuated when compared with a *C. jejuni* wild-type isolate. The piglets inoculated with the *C. jejuni ciaB* null mutant did not develop diarrhoea until 3 days post inoculation whereas all piglets inoculated with a *C. jejuni* wild-type isolate developed diarrhoea within 24 h (Raphael *et al*., 2005).

Gram-negative bacteria have evolved distinct secretion systems to actively transport proteins across their membranes (Thanassi and Hultgren, 2000; Pallen *et al*., 2003; Kostakioti *et al*., 2005). The T3SS is characterized by the export of proteins across both membranes of the bacterium, which normally occurs upon bacteria–host cell contact (Cornelis, 2006; Galan and Wolf-Watz, 2006). In *C. jejuni*, the only T3SS is the flagellar apparatus (Parkhill *et al*., 2000). Previous work has demonstrated that the secretion of *C. jejuni* Cia and other virulence proteins is dependent on a functional flagellar T3SS (Konkel *et al*., 2004; Poly *et al*., 2007). Precedence for the secretion of a virulence factor from the flagellum was first demonstrated with *Yersinia enterocolitica* (Schmiel *et al*., 1998; 2000; Young *et al*., 1999), which utilizes the flagellar T3SS to export a phospholipase termed YplA.

The majority of the proteins secreted from *C. jejuni,* including the Cia virulence proteins, have not yet been identified due in part to low levels of protein secretion under *in vitro* conditions. The aim of this study was to identify a virulence protein that is secreted from the *C. jejuni* flagellar T3SS. As a first step in the identification of putative *Campylobacter-*secreted proteins (Csp), we tested if CiaB would be recognized and secreted from the well-characterized flagellar T3SS of *Y. enterocolitica* (Warren and Young, 2005). Based on the finding that CiaB was secreted from *Y. enterocolitica,* we developed a screen that utilized *Y. enterocolitica* and the YplA effector protein to identify *C. jejuni* genes that encode amino-terminal residues that facilitate protein secretion in a T3SS-dependant manner (i.e. T3S amino-terminal sequences) (Schmiel *et al*., 2000; Berring *et al*., 2004; Warren and Young, 2005). We demonstrated that the screen had the potential to identify putative Csp with T3S amino-terminal sequences using known *C. jejuni* flagellar secreted proteins. We report the identification of 42 *C. jejuni* proteins with amino-terminal sequences that promote secretion from the *Y. enterocolitica* flagellar T3SS. From this list, one gene (*Cj1242*) encoding a hypothetical protein was selected for additional study. We generated a mutation in *Cj1242*, and examined the growth rate, motility, secretion profile and adherence and invasion properties of the *C. jejuni Cj1242* mutant relative to the wild-type isolate. The *C. jejuni Cj1242* mutant displayed an altered secretion profile and reduced host cell invasion, demonstrating that Cj1242 is a virulence protein.

## Results

### The CiaB protein is secreted via the *Y. enterocolitica* flagellar T3SS

**Fig. 1:**
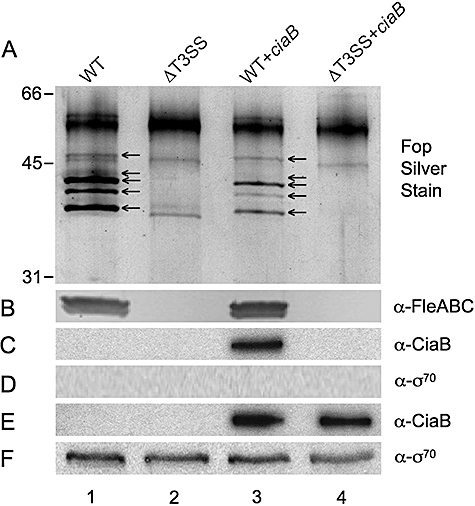
The *C. jejuni* CiaB protein is secreted via the *Y. enterocolitica* flagellar T3SS. Supernatants (A–D) and whole-cell lysates (E and F) were analysed by SDS-PAGE coupled with silver staining or immunoblot analysis. A. Silver stain showing the flagellar outer proteins (Fops) and FleABC. B. Immunoblot probed with the flagellin antibody (FleABC, 38–40 kDa). C. Immunoblot probed with the CiaB antibody (CiaB, 73 kDa). D. Immunoblot probed with the RNA polymerase σ^70^ antibody. E. Immunoblot probed with the CiaB antibody. F. Immunoblot probed with the σ^70^ antibody. Lanes: 1, *Y. enterocolitica* wild-type harbouring the empty pMMB207 vector (WT); 2, *Y. enterocolitica* pYV8081^–^Δ*flhDC ysaT* flagellar mutant harbouring the empty pMMB207 vector (ΔT3SS); 3, *Y. enterocolitica* wild-type harbouring the pMMB207 vector containing the *C. jejuni ciaB* gene (WT + *ciaB*); and 4, *Y. enterocolitica* pYV8081^–^Δ*flhDC ysaT* flagellar mutant harbouring the pMMB207 vector containing the *C. jejuni ciaB* gene (ΔT3SS + *ciaB*).

Based on the finding that CiaB is secreted via the flagellar T3SS of *C. jejuni* (Konkel *et al*., 1999), we reasoned that CiaB should be recognized and secreted in a T3SS-dependent manner in a heterologous system. To test this possibility, the full-length *ciaB* gene was cloned into the pMMB207 plasmid and conjugated into the *Y. enterocolitica* JB580v wild-type strain and *Y. enterocolitica* GY4492, a mutant lacking any functional T3SS (pYV8081^-^Δ*flhDC ysaT*). These bacterial strains and plasmids are described in Table 1. Whole-cell lysate and supernatants were collected from the *Y. enterocolitica* strains cultured under conditions to induce the secretion of the flagellar outer proteins (Fops) (i.e. 2 h at 26°C in TYE broth medium). The Fops represent a set of at least 12 proteins secreted from the flagellar T3SS, including the flagellar filament proteins FleABC. As expected, the *Y. enterocolitica* JB580v wild-type strain secreted the Fops, whereas the *Y. enterocolitica* pYV8081^-^Δ*flhDC ysaT* mutant did not secrete the Fops (Fig. 1A). The supernatants were also probed with the mouse monoclonal flagellin-specific antibody 15D8 for the detection of the *Y. enterocolitica* FleABC flagellar filament proteins (38–40 kDa) (Kapatral and Minnich, 1995). The FleABC proteins were detected in the supernatants of *Y. enterocolitica* JB580v wild-type strain, demonstrating that the flagellar T3SS was functional, whereas the FleABC proteins were not detected from supernatants of the *Y. enterocolitica* pYV8081^-^Δ*flhDC ysaT* T3SS mutant (Fig. 1B). Importantly, the CiaB protein (73 kDa) was detected in the supernatant of the *Y. enterocolitica* JB580v wild-type strain, but not the *Y. enterocolitica* flagellar mutant, as judged by immunoblot analysis with a rabbit polyclonal CiaB-specific antibody (Fig. 1C). The detection of CiaB protein in the supernatant was not due to bacterial cell lysis, as the cytoplasmic protein sigma 70 (σ^70^) was not detected in the supernatants (Fig. 1D). As an additional control, we found that CiaB was synthesized and could be detected in the whole-cell lysate of the *Y. enterocolitica* pYV8081^-^Δ*flhDC ysaT* T3SS mutant (Fig. 1E). As expected, the *Y. enterocolitica* cytoplasmic protein σ^70^ was detected in the whole-cell lysates prepared from each of the bacterial strains (Fig. 1F). Collectively, these results indicate that CiaB is recognized as a flagellar T3 protein secreted by *Y. enterocolitica*.

**Table 1:** Strains and plasmids.

	Genotype	Source or reference
Strains		
*C. jejuni*		
NCTC 11168	Wild-type genome sequenced strain	Parkhill *et al*. (2000)
F38011	Clinical isolate	Rivera-Amill and Konkel (1999)
F38011 *flgB*	Insertion disruption of *flgB*, non-motile and Cia protein secretion deficient	Konkel *et al*. (2004)
*Y. enterocolitica*		
JB580v	Serogroup O:8, Nal *yenR* (r− m+)	Kinder *et al*. (1993)
GY4478	JB580v, pYV8081−	Young and Young (2002)
GY4757	JB580v Δ*yplAB*, pYV8081−	Warren and Young (2005)
GY4492	JB580v Δ*flhDC ysaT*::Tn*Mod*-RKm, pYV8081−	Young and Young (2002)
*Escherichia coli*		
S17-1 lambda *pir*	*recA thi pro hsd*R− M+ RP4::2-Tc::Mu::Km Tn7 *pir*	Simon *et al*. (1983)
Inv-alpha F′	F′*end*A1 *rec*A1 *hsd*R17 (r−, m+) *sup*E44 *thi*-1 *gyr*A96 *rel*A1 f80*lac*ZDM15 D(*lac*ZYA-*arg*F) U169l-	Invitrogen
XL1-Blue	*recA1 endA1 gyrA96 thi-1 hsdR17 supE44 relA1 lac* (F′*proAB lacIZ*Δ*M15* Tn*10*)	Stratagene
Plasmids		
pMMB207	*mob*+, low copy vector containing an inducible *tac* promoter (P*tac*), Cm	Morales *et al*. (1991)
pMEK250	pMMB207 harbouring the full-length 1.9 kb *ciaB* gene driven by P*tac*	This study
pTM100	*mob+,* derivative of pACYC184, Cm Tet	Michiels and Cornelis (1991)
pCSP50	P*cat* upstream of NdeI and BglII sites for directional cloning of fusions with 5′-truncated *yplA* (lacking nucleotides 4–150) and complete *yplB* locus cloned into pTM100 *Eco*RI site, Tet	This study
pCSP50-*yplA* 1–108	Nucleotides 1–108 of *yplA* fused to truncated *yplA* in pCSP50	This study
pCSP50-*flaA* 1–108	Nucleotides 1–108 of *flaA* (Cj1339c) fused to truncated *yplA* in pCSP50	This study
pCSP50-*flaC* 1–108	Nucleotides 1–108 of *flaC* (Cj0720c) fused to truncated *yplA* in pCSP50	This study
pCSP50-*ciaB* 1–108	Nucleotides 1–108 of *ciaB* fused to truncated *yplA* in pCSP50	This study
pCSP50-*cysM* 1–108	Nucleotides 1–108 of *cysM* fused to truncated *yplA* in pCSP50	This study
pBluescript II SK+	Phagemid cloning vector	Stratagene
pMW10	*C. jejuni–E. coli* shuttle vector, Kan	Wosten *et al*. (1998)
pBSK-Kan2	pBluescript II SK+ with original ampicillin cassette replaced by the native promoter and *apha3* gene from pMW10, Kan	This study
pBSK-Kan2:del*Cj1242*	pBSK-Kan2 with *Cj1242* internal deletion and disrupted with *tetO* from pUOA3, Kan Tet	This study
pRY111	*C. jejuni–E. coli* shuttle vector, pWKS29 MCS, Cm	Yao *et al*. (1993)
pRY111:*Cj1242*	pRY111 with a 1.7 kb fragment encompassing *Cj1242*, Cm	This study

### *C. jejuni* T3S amino-terminal sequences promote secretion from the *Y. enterocolitica* flagellar T3SS

**Fig. 2:**
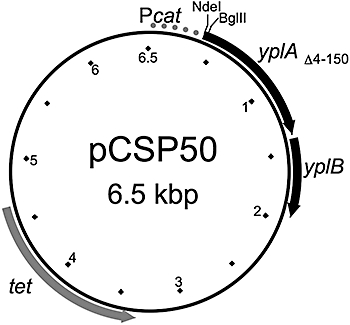
The pCSP50 shuttle vector. The NdeI and BglII sites flank the 5′ end of a truncated *yplA* gene and facilitate directional cloning to generate fusions with 108 bp amino-terminal sequences from *C. jejuni* genes.

All proteins exported via a T3SS contain an amino-terminal sequence to direct their export from the bacterial cell. Moreover, previous work has shown that a T3SS protein can be: (i) recognized and secreted by more than one T3SS in the same bacterium and (ii) recognized and secreted from bacteria that belong to other genera (Young and Young, 2002; Lee and Galan, 2004; Badea *et al*., 2009). As CiaB was secreted via the *C. jejuni* and *Y. enterocolitica* flagellar T3SS, we hypothesized that the CiaB amino-terminus would direct the export of a fusion protein from *Y. enterocolitica* in a T3SS-dependent manner. In addition, we hypothesized that the amino-termini of two other *C. jejuni* flagellar-secreted proteins, FlaA and FlaC, would also promote secretion of a fusion protein. To test this hypothesis, we generated the pCSP50 shuttle vector encoding the *Y. enterocolitica yplA* phospholipase gene as a reporter (Fig. 2). The *Y. enterocolitica* YplA enzyme is an A2 phospholipase and is secreted under flagellar T3SS inducing conditions *in vitro* (Schmiel *et al*., 1998; Berring *et al*., 2004). Warren and Young (2005) determined that the YplA enzyme's T3S amino-terminal sequence is localized within the first 20 residues. The pCSP50 shuttle vector incorporates a constitutive promoter (*cat*) upstream of NdeI and BglII cloning sites, a 5′-truncated *yplA* gene (eliminating the first 50 amino acids including the T3S amino-terminal sequence), and the *yplB* chaperone gene. The amino-terminal deletion of the *Y. enterocolitica* YplA protein abolished its secretion, but not its enzymatic (phospholipase) activity (not shown) (Hatic *et al*., 2002).

**Fig. 3:**
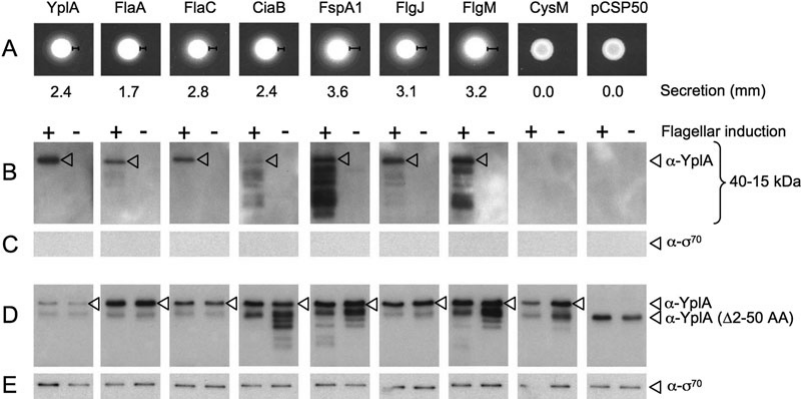
Secretion of YplA fusion proteins from *Y. enterocolitica* under flagellar T3SS-inducing conditions. The first 36 amino acids of each indicated protein was fused to YplA encoded on vector pCSP50. The YplA secretion zone widths (mm) were measured from the edge of the bacterial growth to the outer edge of the fatty acid precipitate. Detection of the YplA fusion protein by immunoblot analysis was done with cultures grown under flagellar-inducing conditions (lanes marked ‘+’) and non-inducing conditions (lanes marked ‘−’). A. A representative PLA assay is shown indicating YplA fusion proteins and secretion zone widths. B. Immunoblot analysis of supernatants probed with the YplA antibody. C. Immunoblot analysis of supernatants probed with the RNA polymerase σ^70^ antibody. D. Immunoblot analysis of whole-cell lysates probed with the YplA antibody. E. Immunoblot analysis of whole-cell lysates probed with the σ^70^ antibody.

The *Y. enterocolitica* JB580v wild-type strain secretes YplA under flagellar T3SS-inducing conditions and the enzymatic activity can be detected on phospholipase agar (PLA) plates (not shown). The hydrolysis of Tween 80 in PLA plates results in a fatty acid precipitate that forms a halo surrounding the YplA secretion-competent colonies. In contrast, the *Y. enterocolitica yplAB* strain GY4757, generated for use in conjunction with a YplA reporter, showed no detectable phospholipase activity. Similarly, the *Y. enterocolitica yplAB* strain harbouring the native pCSP50 vector was secretion negative (Fig. 3A). However, when the first 108 nucleotides of *yplA* (1–36 AA encoding sequence) was fused to the truncated *yplA* gene, the YplA fusion protein was secreted and detected on PLA plates.

To provide proof of concept for the screen for *C. jejuni* genes harbouring T3S amino-terminal sequences, we generated *yplA* fusions with the first 108 nucleotides of three genes encoding proteins known to be secreted via the *C. jejuni* flagellar T3SS (FlaA, FlaC, CiaB) (Fig. 3A). As predicted, all three fusions with YplA were secreted and detected on PLA plates. In contrast, a fusion of the first 108 nucleotides of the *C. jejuni* gene for CysM was generated to serve as a T3SS negative control, and no secretion was observed. CysM is a 32.4 kDa cytoplasmic protein (*O*-acetylserine sulfhydrylase B) involved in cysteine biosynthesis (Garvis *et al*., 1997).

### Identification of *C. jejuni* genes harbouring putative T3S amino-terminal sequences

The results from the native CiaB secretion assay and the YplA reporter assay demonstrated that the *Y. enterocolitica* flagellar system could be utilized to identify a *C. jejuni* protein with a T3S amino-terminal sequence. Thus, a total of 359 genes from the 1654 identified ORFs from the *C. jejuni* NCTC 11168 sequence were selected to test via the YplA reporter assay (Parkhill *et al*., 2000). These genes/ORFs were chosen for analysis as the deduced amino acid sequences lack predicted membrane-spanning domains, periplasmic domains, Sec-dependent signals or Tat-dependent signals. No genes were found to encode type I Sec-independent motifs. Primers were designed to amplify the first 108 encoding bases of all 359 ORFs and facilitate directional cloning into the shuttle vector pCSP50 to generate translational fusions with the truncated YplA reporter. The first 108 bp for 341 of the 359 ORFs were successfully cloned and sequence confirmed in the *Escherichia coli* S17-1 λ-*pir* donor strain. From this fusion library, 321 vectors were successfully conjugated into the *Y. enterocolitica yplAB* host strain and characterized for YplA secretion on PLA plates (Table S1). Table 2 lists the 42 *C. jejuni* genes that harbour amino-terminal sequences that resulted in YplA secretion zone widths greater than or equal to that obtained with the CiaB amino-terminus from the *Y. enterocolitica yplAB* strain after 12 h incubation on PLA plates.

**Table 2:** *C. jejuni* genes encoding a putative T3S amino-terminal sequence.

Gene^a^	Locus	Product description
*flgM*^b,c^	Cj1464	Anti-sigma 28 factor
*fspA1*^b,e^	Cj0859c	Flagellar secreted protein, virulence factor
*rrc*^b^	Cj0012c	Non-haem iron protein, rubrerythrin
Cj0036	Cj0036	Hypothetical protein
Cj1242^d^	Cj1242	Hypothetical protein
*flgJ*^b,d,f^	Cj1463	Flagellar rod protein
Cj0073c^d^	Cj0073c	Conserved hypothetical protein
Cj0122	Cj0122	Hypothetical protein
Cj0125c	Cj0125c	Hypothetical protein
Cj0140	Cj0140	Hypothetical protein
Cj0239c	Cj0239c	NifU protein homologue
Cj0251c	Cj0251c	Conserved hypothetical protein
Cj0787^d^	Cj0787	Conserved hypothetical protein
Cj0788^d^	Cj0788	Hypothetical protein
Cj0015c	Cj0015c	Hypothetical protein
Cj0021c^b^	Cj0021c	Putative fumarylacetoacetate hydrolase family protein
Cj0030	Cj0030	Hypothetical protein
*hemN*	Cj0363c	Putative oxygen-independent coproporphyrinogen III oxidase
Cj0416	Cj0416	Hypothetical protein
Cj0449c	Cj0449c	Conserved hypothetical protein
Cj0849c	Cj0849c	Conserved hypothetical protein
Cj1300^b^	Cj1300	Putative SAM domain containing methyltransferase
Cj1543^b^	Cj1543	Putative allophanate hydrolase subunit 2
Cj0188c^d^	Cj0188c	Putative kinase
Cj0254	Cj0254	Hypothetical protein
Cj0391c	Cj0391c	Hypothetical protein
Cj0717^b^	Cj0717	Putative ArsC family protein
Cj0973	Cj0973	Hypothetical protein
Cj1006c^b,d^	Cj1006c	Putative MiaB-like tRNA modifying enzyme
Cj1057c^b^	Cj1057c	Putative coiled-coil protein
Cj1089c	Cj1089c	Hypothetical protein
Cj1310c	Cj1310c	Hypothetical protein (617 family)
Cj1348c^b^	Cj1348c	Putative coiled-coil protein
Cj1497c	Cj1497c	Hypothetical protein
Cj0069	Cj0069	Hypothetical protein
Cj0668^b^	Cj0668	Putative ATP/GTP-binding protein
Cj0681	Cj0681	Hypothetical protein
Cj0706^d^	Cj0706	Conserved hypothetical protein
Cj0916c	Cj0916c	Conserved hypothetical protein
Cj1162c^b^	Cj1162c	Putative heavy-metal-associated domain protein
Cj1232	Cj1232	Hypothetical protein
Cj1505c^b^	Cj1505c	Putative two-component response regulator (SirA-like protein)

a *C. jejuni* gene *yplA* fusions with a secretion zone width greater than or equal to the zone obtained for the *ciaB : yplA* fusion strain. Listed in descending order of secretion zone width; ascending locus number for equivalent zones.

b Not annotated in original NCTC 11168 sequence analysis (Parkhill *et al*., 2000).

c (Hendrixson and DiRita, 2003; Wosten *et al*., 2004).

d Upregulated when grown in the presence of DOC (Malik-Kale *et al*., 2008).

e (Poly *et al*., 2007).

f (Pallen *et al*., 2005).

### *C. jejuni*–YplA fusion proteins are secreted to the culture supernatant by the *Y. enterocolitica* flagellar T3SS

To confirm that the YplA fusion enzyme activity measured by the PLA plate assay was the result of secretion through the *Y. enterocolitica* flagellar T3SS, we tested several strains by immunoblot analysis. *Y. enterocolitica* strains harbouring the pCSP50 vector with *C. jejuni* amino-terminal sequences were grown in broth culture under conditions that induced or repressed synthesis of the flagellar system. Importantly, the *C. jejuni* amino-terminal sequences fused to YplA were only detected in the supernatants of strains cultured under flagellar-inducing conditions (Fig. 3B). The amount of protein secreted into the supernatant, as judged by immunoblot analysis was roughly proportional to that measured by the PLA plate assay and varied according to the *C. jejuni* amino-terminal sequence. As predicted from the PLA plate assays, there were no reactive bands detected from the supernatants for the strains harbouring the CysM : YplA fusion or the pCSP50-truncated YplA. To evaluate the supernatants for bacterial lysis, which would result in the release of cytoplasmic proteins, the blots were probed using a mouse monoclonal antibody to the cytoplasmic protein σ^70^. No reactive band was detected for σ^70^ in any supernatants (Fig. 3C). Immunoblot analysis of the whole-cell lysates with a rabbit polyclonal YplA-specific antibody confirmed that the YplA fusion proteins (33.0–33.4 kDa, depending on amino-terminal sequence) were being synthesized under both flagellar and non-flagellar conditions (Fig. 3D). In addition, a band of consistent intensity was detected for both growth conditions corresponding to σ^70^ in the whole-cell lysate samples (Fig. 3E). Cumulatively, these data indicate that the YplA fusion proteins were secreted from the flagellar T3SS.

### Functional classification of *C. jejuni* proteins harbouring putative T3S amino-terminal sequences

The functional classifications of the 42 proteins harbouring putative T3S amino-terminal sequences were obtained from the Sanger Institute website (http://www.sanger.ac.uk/) (Gundogdu *et al*., 2007). The majority of the *C. jejuni* NCTC 11168 proteins were classified as either conserved hypothetical proteins (16 proteins) or proteins of unknown function (14 proteins). Noteworthy is that two flagellar-related proteins (FlgM, FlgJ) and a pathogenicity-related protein (FspA) were identified among the proteins harbouring a T3S amino-terminal sequence, which had not been characterized when this study commenced.

### *Cj1242* is secreted from *C. jejuni*

To confirm that one of the proteins identified using the phospholipase indicator agar assay was secreted from the flagellar T3SS of *C. jejuni*, we generated a *Cj1242* deletion mutant. *Cj1242* was chosen because the Cj1242–YplA fusion protein was highly secreted from *Y. enterocolitica* (Table 2), the gene is predicted to be monocistronic and is upregulated when *C. jejuni* is cultured under conditions that induce virulence genes (Malik-Kale *et al*., 2008). The *Cj1242* gene is capable of encoding a protein with a *M*_r_ 12 164. The growth rate and motility of the *Cj1242* deletion mutant were indistinguishable from that of the *C. jejuni* wild-type strain (not shown). We then performed secretion assays to determine if the *C. jejuni Cj1242* mutant was capable of Cia protein export.

**Fig. 4:**
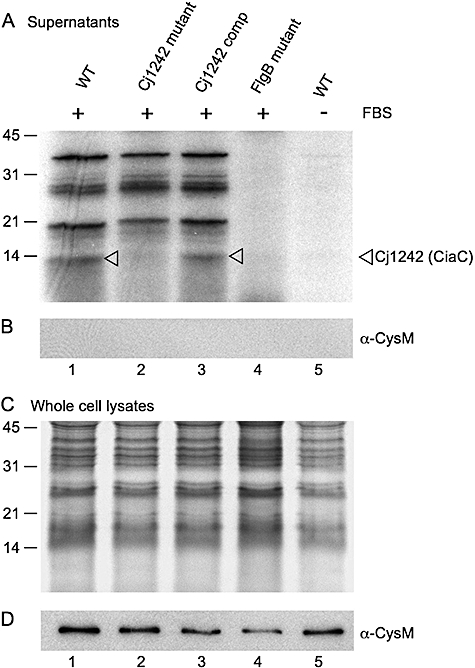
Secretion of Cj1242 (CiaC) from the *C. jejuni* flagellar T3SS. Isolates were incubated in medium containing [^35^S]-methionine and supplemented with 1% FBS or without FBS as described in *Experimental procedures*. Supernatants (A and B) and whole-cell lysates (C and D) were analysed by SDS-PAGE coupled with autoradiography and immunoblot analysis. A. Autoradiograph of supernatant samples; CiaC (12.2 kDa) protein is indicated by an arrowhead. B. Immunoblot of supernatant samples probed with the CysM antibody (32.4 kDa). C. Autoradiograph of whole-cell lysates. D. Immunoblot of whole-cell lysates probed with the CysM antibody. Lanes: (1) *C. jejuni* F38011 wild-type with 1% FBS; (2) *C. jejuni* F38011 *Cj1242* mutant with 1% FBS; (3) *C. jejuni* F38011 *Cj1242* mutant complemented with pRY111 : *Cj1242* with 1% FBS; (4) *C. jejuni* F38011 *flgB* mutant with 1% FBS; (5) *C. jejuni* F38011 wild type without FBS.

The profile of Cia proteins detected from the *C. jejuni* F38011 wild-type strain was similar to that observed in previous work (Konkel *et al*., 2004). In contrast with the wild-type strain, the secretion profile of the *C. jejuni Cj1242* mutant lacked one band of 12.2 kDa (Fig. 4A). The 12.2 kDa band was restored in the *C. jejuni Cj1242* complemented strain, which was transformed with a plasmid harbouring a wild-type copy of *Cj1242 in trans*. Secreted proteins were not detected for the *C. jejuni* wild-type strain when fetal bovine serum (FBS) was omitted from the labelling medium, which is consistent with previous work indicating that components within serum are sufficient to induce Cia protein secretion (Konkel *et al*., 1999; Rivera-Amill *et al*., 2001). In addition, secreted proteins were not detected for the *C. jejuni flgB* mutant incubated with FBS, which is consistent with previous work indicating that Cia protein secretion is dependent on a functional flagellar secretion apparatus (Konkel *et al*., 2004). The presence of the Cia proteins in the supernatants from the wild-type strain, *Cj1242* mutant and *Cj1242*-complemented strain was not due to bacterial lysis, because a 32.4 kDa band was not detected in supernatants probed with the CysM antibody (Fig. 4B). Coomassie brilliant blue (CBB R-250) staining of cell lysates from the secretion assay confirmed that equivalent quantities of protein were loaded (not shown), and an autoradiograph of the dried gel demonstrated equivalent labelling of cellular proteins with [^35^S]-methionine (Fig. 4C). A 32.4 kDa band was detected in the whole-cell lysates with the CysM antibody (Fig. 4D). Cumulatively, these data indicate that Cj1242 (CiaC) was secreted from the flagellar T3S system.

### Cj1242 (CiaC) is required for maximal *C. jejuni* invasion of host cells

Possible differences in bacterial adhesion and invasion between the *C. jejuni* wild-type strain and *Cj1242* mutant were explored by the inoculation of human INT 407 epithelial cells. Quantification of adherent (i.e. cell-associated) and intracellular bacteria by the gentamicin-protection assay revealed that the adherence of the *C. jejuni* wild-type and the *Cj1242* mutant to the INT 407 cells was indistinguishable from one another, but that the *C. jejuni Cj1242* mutant was reduced in host cell invasion when compared with the wild-type isolate (*P* < 0.01) (Table 3). Based on the deficiency in host cell internalization, we designated the protein encoded by *Cj1242* as *Campylobacter* invasion antigen C (CiaC).

**Table 3:** Adherence and internalization of the *C. jejuni* wild-type strain and isogenic mutants.

	Numbers of viable bacteria		
Bacterial strain	Adherent	Internalized	I/A^a^
*C. jejuni* wild-type	(7.1 ± 0.6) × 10^5^	(3.3 ± 0.5) × 10^4^	4.6
*C. jejuni Cj1242*	(7.6 ± 1.2) × 10^5^	(6.2 ± 1.9) × 10^3^	0.82^b^
*C. jejuni ciaB*	(7.2 ± 1.2) × 10^5^	(4.0 ± 0.6) × 10^3^	0.56b
*E. coli* XL1-Blue	(1.8 ± 0.4) × 10^5^	(1.7 ± 1.2) × 10^2^	0.09

a Per cent of internalized bacteria relative to adherent bacteria.

b Internalization of the *C. jejuni Cj1242* and *C. jejuni ciaB* mutants was significantly different from the wild-type strain (*P* < 0.01) as judged by analysis using unpaired Student's *t*-tests.

## Discussion

The goal of this study was to identify a *C. jejuni* secreted protein. To accomplish this goal, we developed a screen using *Y. enterocolitica* to identify genes from *C. jejuni* that contained a T3S amino-terminal sequence. As a first step, we showed that the full-length CiaB protein from *C. jejuni* was synthesized by *Y. enterocolitica* and exported via the flagellar T3SS. We then demonstrated that the amino-terminal sequences of the *C. jejuni* CiaB, FlaA and FlaC proteins were sufficient to drive secretion of a YplA fusion protein from *Y. enterocolitica*. FlaA, FlaC and CiaB proteins are known to be secreted from the *C. jejuni* flagellum (Konkel *et al*., 2004; Song *et al*., 2004). Collectively, these data demonstrate proof of concept for screening *C. jejuni* proteins for T3S amino-terminal sequences using the *Y. enterocolitica* PLA plate assay. We then utilized the assay to test for the presence of T3S amino-terminal sequences in 321 genes from *C. jejuni*. Using the criteria outlined, a total of 42 *C. jejuni* genes were identified that encode amino-terminal sequences that promoted YplA fusion secretion from *Y. enterocolitica* at levels equal to or higher than the CiaB : YplA fusion protein. One of the 42 genes identified was *Cj1242*, which we demonstrate is a potentially important virulence determinant.

While the study was in progress, information on three of the 42 *C. jejuni* proteins identified in the YplA screen was published by other research groups. These studies identified two flagellar-related proteins (FlgM, FlgJ) and a pathogenicity-related protein (FspA1). FlgM (Cj1464) is an anti-sigma factor involved in blocking the promoter binding activity of σ^28^ and the cytoplasmic levels can be controlled by secretion through the flagellar T3SS (Hendrixson and DiRita, 2003; Wosten *et al*., 2004). Although the precise role of FlgJ (Cj1463) in *C. jejuni* is unknown, FlgJ of *Salmonella enterica* is a two-domain protein consisting of an N-terminal domain (including the T3S amino-terminal sequence) involved in flagellar rod formation and a C-terminal region involved in flagellar L ring and hook formation (Nambu *et al*., 1999; Hirano *et al*., 2001). Interestingly, the *C. jejuni* FlgJ protein contains the corresponding N-terminal region as found in other ε-proteobacteria (including *H. pylori*), but it lacks the C-terminal acetylmuramidase region found in most β- and γ-proteobacteria (Pallen *et al*., 2005; Nambu *et al*., 2006). FspA is a 15.5 kDa protein that is secreted from *C. jejuni* via the flagellum (Poly *et al*., 2007). Two variant forms of FspA (A1 and A2) have been identified among *C. jejuni* strains. FspA2 was found to associate with the host cell monolayer and induce apoptosis when added to cell culture in purified form. Validation of the YplA screen described herein lies in the finding that the amino-termini of FlaA, FlaC, CiaB, FlgM, FlgJ and FspA all drive YplA export from *Yersinia* via the flagellar T3SS, whereas fusion of the amino-terminus of a known cytoplasmic protein (CysM) to YplA did not. Importantly, FlaA, FlaC, CiaB, FlgM and FspA all contribute to *C. jejuni* pathogenesis.

Previous work in our laboratory has demonstrated that culturing *C. jejuni* with physiological concentrations of the bile acid deoxycholate (DOC) results in the upregulation of 150 genes (Malik-Kale *et al*., 2008). DOC is also known to induce the synthesis of the *Campylobacter* invasion antigens (Cia) that are secreted via the flagellar T3SS (Konkel and Cieplak, 1992; Konkel *et al*., 1993; 1999; 2004; Rivera-Amill *et al*., 2001). We found that eight of the genes induced by DOC also harbour T3S amino-terminal sequences as judged by PLA plate assay. These genes are of interest because *C. jejuni* cultured in the presence of DOC stimulates this bacterium's pathogenic activity, which is evidenced by an increase in the kinetics of *C. jejuni*-host cell invasion (Malik-Kale *et al*., 2008).

The ultimate goal of this study was to identify a *C. jejuni* Cia virulence protein. The first Cia protein (CiaB) was identified in 1999 (Konkel *et al*., 1999), but the remaining Cia proteins have proven difficult to identify using traditional proteomic approaches, due in part to low levels of protein secretion under *in vitro* conditions. We selected *Cj1242* for further characterization because the Cj1242–YplA fusion protein resulted in a high level of secretion and the gene is upregulated in *C. jejuni* cultured with DOC. We generated a *Cj1242* mutant and then performed growth rate, motility, protein secretion and cell adherence/internalization assays. The *C. jejuni Cj1242* mutant growth rate in Mueller–Hinton (MH) broth and its motility on 0.4% agar were indistinguishable from the *C. jejuni* wild-type strain (not shown). The profile of secreted proteins from the *C. jejuni Cj1242* mutant lacked one band of the mass predicted for the Cj1242 protein (12.2 kDa). We performed adherence and internalization assays with the *Cj1242* mutant and INT 407 cells, and found that there was no significant difference in the adherence of this mutant to INT 407 cells relative to the *C. jejuni* wild-type strain. However, the gentamicin-protection assay revealed the internalization of the *C. jejuni Cj1242* mutant was significantly reduced when compared with the wild-type strain (*P* < 0.01). Based on the deficiency in host cell internalization, we designated the protein encoded by *Cj1242* as *Campylobacter* invasion antigen C (CiaC).

We consider a *C. jejuni* strain yielding a per cent I/A of greater than 1 as both invasive and pathogenic, as inoculation of piglets with these strains results in clinical symptoms that resemble those of human campylobacteriosis, including diarrhoea with blood in the stool (Raphael *et al*., 2005). Inoculation of newborn piglets with *C. jejuni* wild-type strain (secretion-positive isolates) results in more severe disease when compared with a *C. jejuni ciaB* isogenic mutant (i.e. deficient in secretion of all Cia proteins). Noteworthy is that the I/A ratio (i.e. the per cent of adherent bacteria that invade epithelial cells) for the *C. jejuni ciaC* mutant is less than 1 (I/A = 0.82%), which is similar with the *C. jejuni ciaB* mutant (I/A = 0.56%). Based on this invasion ratio, we hypothesize that the *C. jejuni ciaC* mutant (i.e. deficient in secretion of one Cia protein) would also cause less severe disease than a wild-type strain. Our findings indicate that CiaC is required for *C. jejuni* to efficiently invade epithelial cells, and invasion is a virulence attribute of strains known to cause severe campylobacteriosis.

Analysis of the deduced amino acid sequences of the *C. jejuni* proteins found to harbour a putative T3S amino-terminal sequence revealed some additional information. We utilized two recently developed programs for prediction of T3S proteins (Arnold *et al*., 2009; Löwer and Schneider, 2009) to analyse the *C. jejuni* proteins for T3 amino-terminal sequences and compare the results with our YplA fusion data. While the results were slightly different for each program, at most only 10.6% of the 321 *C. jejuni* proteins tested via the YplA reporter assay are predicted to be secreted. In contrast, 14 of the 42 (33.3%) *C. jejuni* proteins listed in Table 2 are predicted to be secreted by one or both of the prediction programs. Of interest, both algorithms predicted CiaC to be secreted, but neither predicted FlaA and CiaB to be secreted. The failure of these programs to identify known *C. jejuni* flagellar-secreted proteins, including FlaA and CiaB, highlight the need for experimental validation of prediction algorithms.

The deduced amino sequences of two of the 42 proteins contain domains that suggest that they could be localized to the cytoplasm. Cj0012c is annotated as ruberythrin, a protein that protects against oxidative stress (Sztukowska *et al*., 2002; Mydel *et al*., 2006). The amino-terminus (i.e. 36 amino acids) of Cj0012c contains a small non-haeme iron domain found in the desulforedoxin and desulfoferrodoxin proteins of some methanogens and sulphate/sulphur reducers (Marchler-Bauer *et al*., 2007). Cj0363c is annotated as a putative oxidoreductase by inclusion in the cluster of an orthologous group (COG0635) for oxygen-independent coproporphyrinogen III oxidase (*hemN*). Noteworthy is that Cj0363c is distantly related to other proteins in COG0635C (Cj0363c, Cj0580c and Cj0992c) and it does not reside in the vicinity of other *hem* cluster genes on the *C. jejuni* chromosome. Moreover, the predicted products of *Cj0363c*, *Cj0580c* and *Cj0992c* contain a radical S-adenosylmethionine (SAM) domain. Radical SAM proteins catalyse diverse reactions, including methylation, isomerization, sulphur insertion, ring formation, anaerobic oxidation and protein radical formation. Evidence exists that these proteins generate radical species by reductive cleavage of SAM through an unusual iron-sulphur centre (Sofia *et al*., 2001). Although there is no experimental evidence indicating the cellular localization of either Cj0012c or Cj0363c in *C. jejuni,* these two examples highlight the need to analyse each putative T3S protein identified in our screen. It is possible that some of the genes identified using the PLA plate assay may not possess functional T3S amino-terminal sequences recognized in *C. jejuni,* or the amino-terminal region may be folded and/or inaccessible in the native protein. However, recent work also indicates that some bacteria secrete virulence proteins that were previously believed to be located solely in the cytosol (Boel *et al*., 2005).

*Campylobacter jejuni* harbours only one T3SS, the flagellum. As a first step in the identification of a *C. jejuni* virulence protein, we sought to identify genes from *C. jejuni* that harbour T3S amino-terminal sequences that direct their export from the flagellum. We report 42 *C. jejuni* proteins with putative T3S amino-terminal sequences. Moreover, we demonstrated that a mutation in one previously uncharacterized *C. jejuni* gene, *Cj1242*, resulted in an isolate with an altered secretion profile and reduced host cell invasion. We have also demonstrated that the secretion of CiaC is dependent upon a functional flagellar apparatus, which serves to further highlight the importance of the flagellar secretion system in the export of *C. jejuni* virulence proteins. We are currently investigating whether the other proteins identified in this study are secreted from *C. jejuni* and contribute to pathogenesis. The phospholipase reporter assay described herein demonstrates that there is a remarkable level of conservation in T3SS protein recognition among the proteobacteria; *C. jejuni* is a member of the delta-epsilon subdivision of proteobacteria and *Y. enterocolitica* is a member of the gamma subdivision. Based on this finding, we submit that the phospholipase reporter system can be used to identify genes harbouring T3S amino-terminal sequences from a variety of bacteria that possess less well-characterized T3SS.

## Experimental procedures

### Bacterial strains, plasmids and media

The bacterial strains and plasmids are described in Table 1. All *Y. enterocolitica* strains used in this study were derived from strain JB580v (Kinder *et al*., 1993). *C. jejuni* strains were cultured with MH broth or agar supplemented with 5% citrated bovine blood and incubated at 37°C under microaerobic conditions (5% O_2_, 10% CO_2_, 85% N_2_) with chloramphenicol (Cm, 8 μg ml^−1^), kanamycin (Kan, 50 μg ml^−1^) or tetracycline (Tet, 2 μg ml^−1^). *E. coli* strains were cultured at 37°C with Luria–Bertani (LB) broth or agar with Cm (15 μg ml^−1^), Kan (50 μg ml^−1^) or Tet (15 μg ml^−1^). *Y. enterocolitica* strains were incubated at 26°C in LB broth or agar supplemented with Cm (10 μg ml^−1^), nalidixic acid (Nal, 20 μg ml^−1^) or Tet (10 μg ml^−1^).

### *C. jejuni* gene selection for T3S amino-terminal sequence screen

We selected genes to screen for T3S amino-terminal sequences from the original annotation of the *C. jejuni* NCTC 11168 sequence (Parkhill *et al*., 2000). Of 1654 ORFs, 359 were chosen for analysis following the elimination of genes encoding proteins with known functions or containing membrane-spanning domains, periplasmic domains, Sec-dependent signals or Tat-dependent signals. No genes were identified with known type I Sec-independent motifs.

### Recombinant DNA procedures with the pMMB207 and pCSP vectors

Vector pMMB207, harbouring a 1.9 kb fragment encompassing the full-length *ciaB* gene, was PCR-amplified from *C. jejuni* NCTC11168 chromosomal DNA using primers CiaB-F1 (5′-GGA TCC AAA GTT AAA AAG GAG AAT AAA AGT ATG) and CiaB-R1 (5′-TTA TTT TTT CTT ATA TCT TTC AAA TTC TC). Correct orientation of the *ciaB* gene was determined by inducing expression from the P*tac* promoter with 5 mM isopropyl β-D-1-thiogalactopyranoside. Constructs were confirmed by DNA sequencing and conjugated into the *Y. enterocolitica* wild-type and mutant strains.

To facilitate the identification of *C. jejuni* genes that harbour T3S amino-terminal sequences, the pCSP50 shuttle vector was generated. The pCSP50 vector includes a *tet* cassette, a constitutive promoter (*cat*), a 5′-truncated *yplA* gene (lacking 150 nucleotides encoding the native T3S amino-terminal sequence) and the *yplB* gene (cognate chaperone). The NdeI and BglII sites facilitated directional cloning of *C. jejuni* sequences as fusions with the truncated *yplA*. The first 108 bp of the amino-terminal regions of 328 *C. jejuni* genes were PCR-amplified with primers containing restriction sites for directional cloning into pCSP50. The amplicons and pCSP50 vector were digested with NdeI and BglII, DNA fragments ligated and *E. coli* S17-1 λ-*pir* was transformed with Tet selection. Cloned *C. jejuni* gene fragments were confirmed by PCR fragment size and sequence analysis. Vectors were conjugated into *Y. enterocolitica* strains and confirmed by agarose gel electrophoresis of restriction digested plasmid preparations.

### Phospholipase indicator agar assay and analysis

Medium for detecting secretion of the YplA phospholipase and YplA fusion proteins from *Y. enterocolitica* was prepared as described previously (Young and Young, 2002). *Y. enterocolitica* strains were incubated overnight in LB broth with shaking at 26°C. Fop secretion was induced by spotting 1.5 μl of culture on TYE PLA medium (1% tryptone, 0.5% yeast extract, 1.5% agar, 1% Tween 80 and 1 mM CaCl_2_), and incubation at 26°C. Each isolate was tested for secretion at least three times from at least two independent PLA plate assays to ensure reproducible results. The conjugates were tested on PLA plates in groups of 16 in addition to a *Y. enterocolitica* strain expressing wild-type YplA as a positive control. All plates were scanned at 300 dpi resolution (12, 24 and 48 h) to create a digital archive of the secretion results. The secretion zone widths were measured manually from digital images using select tools in Adobe Photoshop CS2 version 9.0.2 (Adobe Systems Incorporated, USA). The 24 h secretion zone widths for the positive controls were consistent for all PLA plates (*n* = 22, average = 3.3 mm, standard deviation = 0.12 mm).

### Rabbit antibodies to YplA and CysM

Polyclonal antibodies against recombinant YplA and recombinant CysM were produced in female New Zealand White rabbits by subcutaneous injection of 100 μg of the immunogens in TiterMax Gold (Sigma). Subsequent booster injections of 50 μg of the immunogens in Freund's incomplete adjuvant were administered 2 and 4 weeks after the primary immunizations. Blood was collected from the rabbits by terminal bleeds. The sera were processed and stored at −80°C. Antibody generation in the New Zealand White rabbits was performed using a protocol approved by the Institutional Animal Care and Use Committee (IACUC protocol #2433) at Washington State University.

### Determination of Fop and YplA fusion protein secretion by immunoblot

*Yersinia enterocolitica* strains were incubated overnight in LB broth with shaking at 26°C. Fop secretion was induced by inoculation of TYE broth (1% tryptone, 0.5% yeast extract) with 1× TYE broth-washed *Y. enterocolitica* cultures and incubation with shaking at 26°C for 4–6 h. The OD_540_ of all cultures was determined, the cells washed 1× with TYE to remove secreted proteins and suspended in fresh TYE at an OD_540_ of 0.5 for the 0 h time point of the secretion assay. After 2 h of shaking at 26°C, OD_540_ were determined for normalization of whole-cell lysate samples, and 1 ml of each supernatant harvested by filtration through 0.22 μm sterile filters. Secreted proteins were precipitated by addition of 111 μl of 6.1 N trichloroacetic acid (10% vol vol^−1^ TCA final), minimum of 1 h incubation at −20°C and centrifugation with two acetone washes. Precipitated proteins were dissolved in 50 μl of single-strength electrophoresis sample buffer and heated to 95°C for 5 min. Proteins were separated by sodium dodecyl sulphate-polyacrylamide gel electrophoresis (12% polyacrylamide) SDS-PAGE with the discontinuous buffer system described by Laemmli (1970). The proteins were electrophoretically transferred to polyvinylidene fluoride membranes (Immobilon P; Millipore Corp., Bedford, MA) for immunoblot analysis. Bound antibodies were detected with peroxidase-conjugated goat anti-rabbit immunoglobulin G or peroxidase-conjugated goat anti-mouse immunoglobulin G. Immunoblot development was done by chemiluminescence (Western Lightning, PerkinElmer Life Sciences) and film exposure (Biomax MR film, Kodak).

### Generation of the *C. jejuni Cj1242* deletion mutant and complement strain

The *Cj1242* gene was disrupted by homologous recombination between the disrupted *Cj1242* gene on a suicide vector and the *Cj1242* gene in the chromosome. The *Cj1242* gene on the suicide vector had been disrupted by insertion of a TetO cassette as outlined below. A 900-base-pair fragment upstream of the *C. jejuni* F38011 *Cj1242* gene was amplified using the primers Cj1242F1SstI (5′-TTG AGC TCG CTC TAG CTA TAA TGG TCA CAG) and Cj1242R1SstII (5′-AAC CGC GGC ATT TGA TGT TTT TTG AGT ATT ATC) and cloned into the pCR2.1 cloning vector (TA cloning system; Invitrogen) as outline by the supplier. An 806-base-pair fragment downstream of the *Cj1242* gene was amplified using the primers Cj1242F2SstII (5′-TTC CGC GGA CTT CGG CAG ATG AAT TTC AAG) and Cj1242R2XhoI (5′-AAC TCG AGG TAA GCT TTA AGG CAT CAT AGA C) and cloned into a separate pCR2.1 cloning vector. The upstream fragment was then restriction-digested with SstI and SstII, gel-purified and ligated into the pCR2.1 cloning vector harbouring the downstream fragment. A 2.4 kb TetO cassette was amplified from pUOA3 (Taylor *et al*., 1987) with primers containing SstII sites and cloned into the SstII site of the pCR2.1 *Cj1242* construct. The resultant 4.1 kb insert was then excised by SstI and XhoI restriction digest, gel-purified and ligated into pBSK-Kan2. The resultant suicide vector was sequence-confirmed and electroporated into the *C. jejuni* F38011 isolate. Transformants were selected on MH blood agar containing Tet 2 μg ml^−1^. Tet-resistant isolates were screened for Kan sensitivity, indicating a double-crossover homologous recombination event and loss of the suicide vector. Tet cassette integration into the *C. jejuni Cj1242* gene was confirmed by PCR.

Construction of a complementation vector for the *Cj1242* gene was accomplished by cloning a PCR product obtained with primers Cj1242F1SstI and Cj1242R2XhoI. The 1.7 kb amplicon encompassing *Cj1242* was digested with SstI and XhoI, gel-purified and ligated into shuttle vector pRY111. The resultant pRY111 : *Cj1242* complementation vector was sequence-confirmed and electroporated into the *C. jejuni* F38011 wild-type strain. Transformants were selected on MH blood agar containing Cm 8 μg ml^−1^ and presence of the vector encoded copy of *Cj1242* was confirmed by PCR.

### INT 407 cell adherence and internalization assays

A stock culture of INT 407 cells (human embryonic intestine, ATCC CCL 6) was obtained from the American Type Culture Collection. The cells were cultured in MEM supplemented with 10% FBS at 37°C in a humidified, 5% CO_2_ incubator. The day prior to an assay, each well of a 24-well tissue culture tray was seeded with 1.5 × 10^5^ cells and incubated for 18 h at 37°C in a humidified, 5% CO_2_ incubator. The following day, the cells were rinsed with MEM-1% FBS and inoculated with approximately 5 × 10^7^ cfu of a bacterial suspension. The tissue culture trays were centrifuged at 600 *g* for 5 min to promote bacteria–host cell contact, and incubated at 37°C in a humidified, 5% CO_2_ incubator. For the adherence assays, the plates were incubated for 30 min. The cell monolayers were then rinsed three times with PBS, epithelial cells lysed with a solution of 0.1% (vol vol^−1^) Triton X-100 (Calbiochem, La Jollo, CA) and bacterial suspensions were serially diluted and spread onto MH blood plates. The number of viable, adherent bacteria was determined by counting the resultant colonies. To assess bacterial internalization, the inoculated cell monolayers were incubated for 3 h, rinsed three times with MEM-1% FBS, and incubated for an additional 3 h in MEM-1% FBS containing a bactericidal concentration of gentamicin (250 μg ml^−1^). The number of internalized bacteria was then determined as outlined above for the adherence assays. The reported values represent the mean counts ± standard deviations derived from triplicate wells. All assays in this study were repeated a minimum of three times to ensure reproducibility and performed at a multiplicity of infection between 50 and 500. Regardless of the multiplicity of infection, the phenotype of the *C. jejuni Cj1242* mutant relative to the wild-type strain was always the same.

### *C. jejuni* secretion assay

The *C. jejuni* F38011 strain and isogenic *Cj1242* mutant was metabolically labelled with [^35^S]-methionine as described elsewhere (Konkel and Cieplak, 1992). Briefly, isolates were harvested from biphasic culture on MH agar supplemented with 0.1% DOC and resuspended in MEM lacking methionine supplemented with or without dialysed albumin depleted FBS to an OD_540_ = 0.3. [^35^S]-methionine was then added and inocula were incubated at 37°C for 3 h under microaerophilic conditions. After incubation, supernatant fluids were concentrated 10-fold by precipitation with 4 vols of ice-cold 1 mM HCl-acetone. The pellets were air-dried and dissolved in an equal amount of water and double-strength sample buffer. Equal volumes of the concentrated samples were subjected to 12% SDS-PAGE. The gel was dried, exposed to film for 5 days, and developed to acquire the auroradiograph.

### Bioinformatics

Operon and regulon prediction was performed by query of the MicrobesOnline site (Alm *et al*., 2005). *In silico* T3S protein prediction was performed using ‘EffectiveT3’ (http://www.chlamydiadb.org; Arnold *et al*., 2009) and ‘Modlab’ software (http://gecco.org.chemie.uni-frankfurt.de/index.html; Löwer and Schneider, 2009).
