# Supplementary material for: Identification of a Campylobacter jejuni-secreted protein required for maximal invasion of host cells
Source: Mol Microbiol. 2009 Jul 27;73(4):650–62. doi: 10.1111/j.1365-2958.2009.06797.x (PMC2764114; doi:10.1111/j.1365-2958.2009.06797.x)
Supplement: Supplementary file 1 [file mmi0073-0650-SD1.pdf]

Table S1. *C. jejuni* genes screened for secretion as *yplA* fusions

Gene	Locus	Product (Gundogdu *et al*., 2007)
rrc	Cj0012c	non-haem iron protein
Cj0015c	Cj0015c	hypothetical protein
dba	Cj0018c	disulphide bond formation protein
Cj0021c	Cj0021c	putative fumarylacetoacetate (FAA) hydrolase family protein
thyX	Cj0026c	thymidylate synthase
Cj0030	Cj0030	hypothetical protein
Cj0036	Cj0036	hypothetical protein
Cj0038c	Cj0038c	putative poly(A) polymerase family protein
Cj0040	Cj0040	hypothetical protein
fliK	Cj0041	putative flagellar hook-length control protein
Cj0044c	Cj0044c	hypothetical protein
Cj0045c	Cj0045c	putative iron-binding protein
Cj0054c	Cj0054c	putative lysine decarboxylase family protein
Cj0062c	Cj0062c	putative integral membrane protein
Cj0067	Cj0067	putative amidohydrolase family protein
Cj0069	Cj0069	hypothetical protein
Cj0073c	Cj0073c	hypothetical protein
Cj0074c	Cj0074c	putative iron-sulfur protein
Cj0075c	Cj0075c	putative oxidoreductase iron-sulfur subunit
Cj0085c	Cj0085c	putative amino acid recemase
Cj0118	Cj0118	hypothetical protein
Cj0120	Cj0120	hypothetical protein
Cj0121	Cj0121	hypothetical protein
Cj0122	Cj0122	hypothetical protein
Cj0125c	Cj0125c	hypothetical protein
Cj0126c	Cj0126c	hypothetical protein
Cj0128c	Cj0128c	putative inositol monophosphatase family protein
Cj0133	Cj0133	putative glycoprotease family protein
Cj0135	Cj0135	hypothetical protein
Cj0138	Cj0138	hypothetical protein
Cj0140	Cj0140	hypothetical protein
Cj0148c	Cj0148c	hypothetical protein
Cj0152c	Cj0152c	putative membrane protein
Cj0156c	Cj0156c	hypothetical protein
Cj0157c	Cj0157c	putative integral membrane protein
Cj0159c	Cj0159c	putative 6-pyruvoyl tetrahydropterin synthase
Cj0160c	Cj0160c	putative radical SAM domain protein
Cj0162c	Cj0162c	putative periplasmic protein
Cj0163c	Cj0163c	hypothetical protein
Cj0172c	Cj0172c	putative saccharopine dehydrogenase
Cj0184c	Cj0184c	possible serine\threonine protein phosphatase
Cj0188c	Cj0188c	putative kinase
Cj0189c	Cj0189c	hypothetical protein
Cj0229	Cj0229	putative acetyltransferase
Cj0230c	Cj0230c	putative transferase protein
Cj0232c	Cj0232c	putative integral membrane protein
Cj0239c	Cj0239c	nifU protein homolog
Cj0241c	Cj0241c	putative iron-binding protein
Cj0249	Cj0249	hypothetical protein
Cj0251c	Cj0251c	highly acidic protein
Cj0253	Cj0253	hypothetical protein
Cj0254	Cj0254	hypothetical protein

Cj0258	Cj0258	putative bacterial regulatory protein, ArsR family
Cj0270	Cj0270	putative tautomerase family protein
Cj0272	Cj0272	hypothetical protein
Cj0286c	Cj0286c	hypothetical protein
Cj0323	Cj0323	hypothetical protein
Cj0327	Cj0327	putative endoribonuclease L-PSP family protein
Cj0331c	Cj0331c	hypothetical protein
Cj0340	Cj0340	putative nucleoside hydrolase
Cj0344	Cj0344	hypothetical protein
Cj0353c	Cj0353c	phosphatase
glmM	Cj0360	phosphoglucosamine mutase
hemN	Cj0363c	putative oxygen-independent coproporphyrinogen III oxidase
Cj0364	Cj0364	hypothetical protein
Cj0372	Cj0372	putative glutathionylspermidine synthase
Cj0373	Cj0373	putative D-2-hydroxyacid dehydrogenase
Cj0374	Cj0374	hypothetical protein
Cj0377	Cj0377	probable AAA family ATPase
Cj0380c	Cj0380c	hypothetical protein
Cj0386	Cj0386	putative GTP-binding protein
Cj0391c	Cj0391c	hypothetical protein
mqo	Cj0393c	putative malate:quinone oxidoreductase
Cj0394c	Cj0394c	putative transcriptional activator
Cj0395c	Cj0395c	hypothetical protein
Cj0397c	Cj0397c	hypothetical protein
Cj0403	Cj0403	hypothetical protein
Cj0404	Cj0404	putative transmembrane protein
Cj0411	Cj0411	putative ATP/GTP binding protein
Cj0412	Cj0412	putative ATP/GTP binding protein
Cj0415	Cj0415	putative GMC oxidoreductase subunit
Cj0416	Cj0416	hypothetical protein
Cj0421c	Cj0421c	putative integral membrane protein
Cj0427	Cj0427	hypothetical protein
Cj0429c	Cj0429c	hypothetical protein
Cj0436	Cj0436	putative pyridoxamine 5'-phosphate oxidase
Cj0447	Cj0447	putative NUDIX hydrolase family protein
Cj0449c	Cj0449c	hypothetical protein
Cj0456c	Cj0456c	hypothetical protein
Cj0457c	Cj0457c	putative lipoprotein
Cj0462	Cj0462	putative radical SAM domain protein
Cj0463	Cj0463	zinc protease-like protein
ctb	Cj0465c	group III truncated haemoglobin
dapA	Cj0481	putative dihydrodipicolinate synthase
Cj0485	Cj0485	putative oxidoreductase
Cj0487	Cj0487	putative amidohydrolase
Cj0488	Cj0488	hypothetical protein
Cj0496	Cj0496	hypothetical protein
Cj0499	Cj0499	putative histidine triad (HIT) family protein
Cj0500	Cj0500	putative rhodanese-like domain protein
Cj0504c	Cj0504c	putative oxidoreductase
Cj0505c	Cj0505c	putative aminotransferase (degT family)
Cj0510c	Cj0510c	hypothetical protein
Cj0519	Cj0519	putative rhodanese-like domain protein
Cj0539	Cj0539	hypothetical protein
ubiD	Cj0546	putative 3-octaprenyl-4-hydroxybenzoate carboxy-lyase
Cj0550	Cj0550	hypothetical protein
Cj0556	Cj0556	putative amidohydrolase family protein

Cj0559	Cj0559	putative pyridine nucleotide-disulphide oxidoreductase
Cj0563	Cj0563	hypothetical protein
Cj0571	Cj0571	putative transcriptional regulator
Cj0573	Cj0573	putative GatB/Yqey family protein
nudH	Cj0581	putative NUDIX hydrolase family protein
Cj0583	Cj0583	hypothetical protein
Cj0600	Cj0600	hypothetical protein
Cj0602c	Cj0602c	MOSC-domain containing protein
Cj0604	Cj0604	putative polyphosphate kinase
Cj0611c	Cj0611c	putative acyltransferase family protein
Cj0620	Cj0620	hypothetical protein
Cj0621	Cj0621	hypothetical protein
holA	Cj0630c	putative DNA polymerase III, delta subunit
Cj0635	Cj0635	putative holliday junction resolvase
Cj0636	Cj0636	NOL1\NOP2\sun family protein
Cj0641	Cj0641	putative inorganic polyphosphate/ATP-NAD kinase
Cj0644	Cj0644	putative TatD-related deoxyribonuclease protein
Cj0647	Cj0647	putative HAD-superfamily hydrolase
Cj0650	Cj0650	putative ATP/GTP binding protein
Cj0667	Cj0667	putative S4 domain protein
Cj0668	Cj0668	putative ATP/GTP-binding protein
Cj0681	Cj0681	hypothetical protein
Cj0682	Cj0682	hypothetical protein
Cj0700	Cj0700	hypothetical protein
Cj0701	Cj0701	putative protease
Cj0703	Cj0703	hypothetical protein
Cj0706	Cj0706	hypothetical protein
Cj0708	Cj0708	putative ribosomal pseudouridine synthase
Cj0711	Cj0711	hypothetical protein
Cj0716	Cj0716	putative phospho-2-dehydro-3-deoxyheptonate aldolase
Cj0717	Cj0717	putative ArsC family protein
Cj0719c	Cj0719c	hypothetical protein
Cj0724	Cj0724	hypothetical protein
Cj0729	Cj0729	putative type I phosphodiesterase/nucleotide pyrophosphatase
Cj0733	Cj0733	putative HAD-superfamily hydrolase
Cj0760	Cj0760	hypothetical protein
Cj0761	Cj0761	hypothetical protein
Cj0786	Cj0786	small hydrophobic protein
Cj0787	Cj0787	hypothetical protein
Cj0788	Cj0788	hypothetical protein
Cj0791c	Cj0791c	putative aminotransferase
Cj0792	Cj0792	hypothetical protein
Cj0794	Cj0794	hypothetical protein
Cj0797c	Cj0797c	hypothetical protein
Cj0800c	Cj0800c	putative ATPase
Cj0805	Cj0805	putative zinc protease
Cj0808c	Cj0808c	small hydrophobic protein
Cj0809c	Cj0809c	putative hydrolase
Cj0823	Cj0823	hypothetical protein
Cj0829c	Cj0829c	putative CoA binding domain containing protein
Cj0833c	Cj0833c	oxidoreductase
Cj0837c	Cj0837c	hypothetical protein
Cj0839c	Cj0839c	hypothetical protein
mobB	Cj0841c	putative molybdopterin-guanine dinucleotide biosynthesis protein
Cj0844c	Cj0844c	putative integral membrane protein
Cj0849c	Cj0849c	hypothetical protein

Cj0852c	Cj0852c	putative integral membrane protein
Cj0859c	Cj0859c	hypothetical protein
Cj0878	Cj0878	hypothetical protein
Cj0880c	Cj0880c	hypothetical protein
Cj0881c	Cj0881c	hypothetical protein
Cj0883c	Cj0883c	putative transcriptional regulator
Cj0898	Cj0898	HIT-family protein
Cj0915	Cj0915	putative hydrolase
Cj0916c	Cj0916c	hypothetical protein
Cj0930	Cj0930	putative GTP-binding protein
Cj0939c	Cj0939c	hypothetical protein
Cj0947c	Cj0947c	putative carbon-nitrogen hydrolase
Cj0949c	Cj0949c	putative peptidyl-arginine deiminase family protein
Cj0954c	Cj0954c	putative dnaJ-like protein
Cj0957c	Cj0957c	hypothetical protein
Cj0959c	Cj0959c	hypothetical protein
Cj0962	Cj0962	putative acetyltransferase
Cj0963	Cj0963	hypothetical protein
Cj0965c	Cj0965c	putative acyl-CoA thioester hydrolase
Cj0971	Cj0971	hypothetical protein
Cj0972	Cj0972	hypothetical protein
Cj0973	Cj0973	hypothetical protein
Cj0976	Cj0976	putative methyltransferase
Cj0977	Cj0977	hypothetical protein
Cj0984	Cj0984	hypothetical protein
Cj0989	Cj0989	putative membrane protein
Cj0990c	Cj0990c	hypothetical protein
Cj0993c	Cj0993c	hypothetical protein
Cj1002c	Cj1002c	putative phosphoglycerate/bisphosphoglycerate mutase
Cj1006c	Cj1006c	putative MiaB-like tRNA modifying enzyme
Cj1009c	Cj1009c	hypothetical protein
Cj1011	Cj1011	putative CorA-like Mg2+ transporter protein
Cj1028c	Cj1028c	possible purine/pyrimidine phosphoribosyltransferase
Cj1034c	Cj1034c	possible dnaJ-like protein
ate	Cj1035c	putative arginyl-tRNA-protein transferase
Cj1036c	Cj1036c	hypothetical protein
Cj1056c	Cj1056c	putative carbon-nitrogen hydrolase family protein
Cj1057c	Cj1057c	putative coiled-coil protein
Cj1063	Cj1063	possible acetyltransferase
Cj1075	Cj1075	hypothetical protein
Cj1084c	Cj1084c	putative ATP/GTP-binding protein
Cj1086c	Cj1086c	hypothetical protein
Cj1089c	Cj1089c	hypothetical protein
lnt	Cj1095	putative apolipoprotein N-acyltransferase
Cj1100	Cj1100	hypothetical protein
clpS	Cj1107	ATP-dependent Clp protease adaptor protein
Cj1112c	Cj1112c	putative SelR domain containing protein
Cj1113	Cj1113	hypothetical protein
Cj1115c	Cj1115c	putative phosphatidylserine decarboxylase-related protein
Cj1144c	Cj1144c	hypothetical protein
gmhB	Cj1152c	D,D-heptose 1,7-bisphosphate phosphatase
Cj1162c	Cj1162c	putative heavy-metal-associated domain protein
Cj1164c	Cj1164c	hypothetical protein
Cj1172c	Cj1172c	hypothetical protein
tatA	Cj1176c	Sec-independent protein translocase (TatA/E homolog)
Cj1178c	Cj1178c	highly acidic protein

Cj1199	Cj1199	putative iron/ascorbate-dependent oxidoreductase
Cj1203c	Cj1203c	putative integral membrane protein
Cj1208	Cj1208	putative 5-formyltetrahydrofolate cyclo-ligase family protein
Cj1211	Cj1211	putative competence family protein
Cj1216c	Cj1216c	hypothetical protein
Cj1217c	Cj1217c	hypothetical protein
Cj1224	Cj1224	putative iron-binding protein
Cj1225	Cj1225	hypothetical protein
Cj1232	Cj1232	hypothetical protein
Cj1233	Cj1233	putative HAD-superfamily hydrolase
Cj1236	Cj1236	hypothetical protein
Cj1237c	Cj1237c	possible phosphatase
Cj1242	Cj1242	hypothetical protein
Cj1245c	Cj1245c	putative membrane protein
Cj1247c	Cj1247c	hypothetical protein
Cj1249	Cj1249	hypothetical protein
Cj1251	Cj1251	hypothetical protein
Cj1254	Cj1254	hypothetical protein
Cj1256c	Cj1256c	putative membrane protein
Cj1268c	Cj1268c	putative FAD dependent oxidoreductase
Cj1270c	Cj1270c	putative 2-nitropropane dioxygenase, oxidoreductase protein
Cj1285c	Cj1285c	hypothetical protein
pseC	Cj1294	C4 aminotransferase specific for PseB product
Cj1295	Cj1295	hypothetical protein
Cj1300	Cj1300	putative SAM domain containing methyltransferase
Cj1305c	Cj1305c	hypothetical protein Cj1305c (617 family)
Cj1306c	Cj1306c	hypothetical protein Cj1306c (617 family)
Cj1307	Cj1307	putative amino acid activating enzyme
Cj1309c	Cj1309c	hypothetical protein
Cj1310c	Cj1310c	hypothetical protein Cj1310c (617 family)
maf3	Cj1334	(Motility accessory factor, function unknown)
maf4	Cj1335	hypothetical protein Cj1335 (1318 family)
pseE/maf5	Cj1337	hypothetical protein
Cj1342c	Cj1342c	(Motility accessory factor, function unknown)
Cj1348c	Cj1348c	putative coiled-coil protein
Cj1349c	Cj1349c	possible fibronectin/fibrinogen-binding protein
Cj1360c	Cj1360c	putative proteolysis tag for 10Sa_RNA
Cj1361c	Cj1361c	hypothetical protein
Cj1367c	Cj1367c	possible nucleotidyltransferase
Cj1370	Cj1370	putative nucleotide phosphoribosyltransferase
Cj1374c	Cj1374c	hypothetical protein
Cj1377c	Cj1377c	putative ferredoxin
Cj1383c	Cj1383c	hypothetical protein
Cj1384c	Cj1384c	hypothetical protein
Cj1386	Cj1386	ankyrin-repeat containing protein
Cj1397	Cj1397	putative ferrous iron transport protein
nadD	Cj1404	putative nicotinate-nucleotide adenylyltransferase
Cj1405	Cj1405	hypothetical protein
Cj1449c	Cj1449c	hypothetical protein
Cj1450	Cj1450	putative ATP/GTP-binding protein
tilS	Cj1453c	putative tRNA(Ile)-lysidine synthase
truD	Cj1457c	tRNA pseudouridine synthase D
Cj1459	Cj1459	hypothetical protein
Cj1460	Cj1460	hypothetical protein
flgJ	Cj1463	hypothetical protein
flgM	Cj1464	hypothetical protein

flgN	Cj1465	hypothetical protein
Cj1467	Cj1467	hypothetical protein
Cj1476c	Cj1476c	pyruvate-flavodoxin oxidoreductase
Cj1477c	Cj1477c	putative hydrolase
Cj1482c	Cj1482c	hypothetical protein
Cj1493c	Cj1493c	putative integral membrane protein
Cj1495c	Cj1495c	hypothetical protein
Cj1496c	Cj1496c	putative periplasmic protein
Cj1497c	Cj1497c	hypothetical protein
Cj1501	Cj1501	hypothetical protein
Cj1505c	Cj1505c	putative two-component response regulator (SirA-like protein)
Cj1507c	Cj1507c	putative regulatory protein
Cj1514c	Cj1514c	hypothetical protein
Cj1521c	Cj1521c	putative CRISPR-associated protein
Cj1522c	Cj1522c	putative CRISPR-associated protein
coaE	Cj1530	putative dephospho-CoA kinase
Cj1533c	Cj1533c	putative helix-turn-helix containsing protein
Cj1534c	Cj1534c	possible bacterioferritin
Cj1541	Cj1541	hypothetical protein
Cj1542	Cj1542	putative allophanate hydrolase subunit 1
Cj1543	Cj1543	putative allophanate hydrolase subunit 2
Cj1548c	Cj1548c	putative NADP-dependent alcohol dehydrogenase
Cj1558	Cj1558	putative membrane protein
Cj1585c	Cj1585c	putative oxidoreductase
Cj1602	Cj1602	hypothetical protein
Cj1613c	Cj1613c	putative pyridoxamine 5'-phosphate oxidase
Cj1627c	Cj1627c	hypothetical protein
Cj1637c	Cj1637c	putative periplasmic protein
Cj1639	Cj1639	nifU protein homolog
Cj1640	Cj1640	hypothetical protein
Cj1642	Cj1642	hypothetical protein
Cj1649	Cj1649	putative lipoprotein
Cj1650	Cj1650	hypothetical protein
Cj1656c	Cj1656c	hypothetical protein
Cj1667c	Cj1667c	repA protein homolog
Cj1671c	Cj1671c	hypothetical protein
Cj1674	Cj1674	hypothetical protein
Cj1679	Cj1679	hypothetical protein
Cj1710c	Cj1710c	putative metallo-beta-lactamase family protein
Cj1712	Cj1712	hypothetical protein
Cj1713	Cj1713	putative radical SAM domain protein
Cj1714	Cj1714	small hydrophobic protein
Cj1715	Cj1715	putative acetyltransferase
Cj1720	Cj1720	hypothetical protein
Cj1724c	Cj1724c	putative GTP cyclohydrolase I
